# Explaining English learning motivation in digital EFL environments through emotional intelligence, academic well-being, and perceived digital learning support

**DOI:** 10.3389/fpsyg.2026.1926040

**Published:** 2026-09-25

**Authors:** Dandan Guo, Jinfang Yao, Hisham Noori Hussain Al-Hashimy

**Affiliations:** 1 Department of General Education, Henan Vocational University of Science and Technology, Zhoukou, Henan, China; 2 School of Business, Huanghe Science and Technology University, Zhengzhou, Henan, China; 3 School of Languages, Literacies and Translation, Universiti Sains Malaysia, George Town, Penang, Malaysia; 4 School of Management, Universiti Sains Malaysia, Penang, Malaysia

**Keywords:** academic well-being, EFL learners, emotional intelligence, English learning motivation, perceived digital learning support

## Abstract

**Introduction:**

The rapid expansion of digital learning environments has transformed English as a Foreign Language (EFL) education and increased the importance of understanding the psychological and contextual factors associated with EFL learners' motivation. This study examined the relationships among Emotional Intelligence (EI), Academic Well-Being (AWB), English Learning Motivation (ELM), and Perceived Digital Learning Support (PDLS) in digitally supported English as a Foreign Language environment.

**Methods:**

A quantitative cross-sectional survey design was employed using 435 valid responses, and the proposed relationships were evaluated through partial least squares structural equation modelling using WarpPLS.

**Results:**

The measurement model demonstrated satisfactory reliability and validity. EI was positively associated with AWB (β = 0.354, *p* < 0.001) and ELM (β = 0.331, *p* < 0.001). AWB was also positively associated with ELM (β = 0.229, *p* < 0.001). The Stable3 output indicated a positive indirect association between EI and ELM through AWB (β = 0.081, *p* = 0.008). It also indicated a positive interaction between PDLS and AWB in relation to ELM (β = 0.180, *p* < 0.001). The model explained 12.5% of the variance in AWB and 24.3% of the variance in ELM.

**Discussion:**

The findings indicate that EI, AWB, and PDLS are positively associated with ELM. However, because the analysis used 100 Stable3 resamples and did not generate conventional bootstrapped confidence intervals, the indirect and interaction results are provisional associations that require confirmation through conventional bootstrapping. Given the cross-sectional design, the findings represent statistical associations rather than causal relationships.

## 1 Introduction

The expansion of digital technologies has substantially reshaped educational systems worldwide, particularly within English as a Foreign Language (EFL) education. Digital learning environments now offer flexible access to instructional resources, interactive platforms, and opportunities for self-directed learning (Jeong, 2022; Lee and Chang, 2025). Although these developments have enhanced accessibility and diversified instructional practices, they have also introduced new psychological demands on English as a Foreign Language (EFL) learners, including reduced face-to-face interaction, increased autonomy, and the need for sustained self-regulation. In this context, identifying the psychological factors that support EFL learners' motivation has become an important concern in educational psychology. Traditionally, English language learning has relied on structured classroom instruction, teacher guidance, and externally driven motivational strategies (Liu et al., 2024). While these approaches continue to play a valuable role, they may not fully address the emotional and cognitive challenges of digital learning environments, where EFL learners must manage their own engagement and learning processes (Ge, 2025). Recent research has highlighted the importance of internal psychological resources, particularly Emotional Intelligence (EI), defined as the capacity to perceive, regulate, and utilize emotions effectively (Hameli et al., 2025). EFL Learners with higher EI are more likely to cope with stress, maintain positive attitudes, and persist in demanding learning situations.

In addition, Academic Well-Being (AWB) is a key factor in educational settings, reflecting EFL learners' positive emotional and cognitive evaluations of their academic experiences, including engagement, satisfaction, and reduced stress (Wong et al., 2024). In digital EFL contexts, where EFL learners may experience limited social interaction and increased independence, AWB becomes particularly relevant. At the same time, English Learning Motivation (ELM) remains a central determinant of successful language learning, influencing EFL learners' effort, persistence, and long-term achievement (Tuan, 2025). Despite growing scholarly interest in EI, AWB, and ELM, several important gaps remain. First, existing studies have largely examined these variables as separate constructs, offering limited understanding of how emotional resources, psychological well-being, and learning motivation interact within a unified explanatory framework. Previous research has established the importance of EI for academic outcomes and well-being (Shengyao et al., 2024), highlighted the role of AWB in EFL learner engagement and academic functioning (Huo, 2022), and emphasized the significance of motivation in language learning success (Wang and Xue, 2022). However, the mechanisms through which these constructs jointly influence language learning outcomes remain insufficiently explained. Second, much of the existing evidence comes from traditional educational settings. The rapid expansion of online and blended learning has transformed English language education and introduced new challenges related to learner autonomy, emotional regulation, and sustained engagement (Chen, 2022). Digital learning environments often require learners to manage their own learning processes while coping with reduced face-to-face interaction and increased demands for self-regulation (Sharma et al., 2024). Consequently, findings derived from conventional classroom contexts may not fully explain motivational processes in digitally mediated EFL environments. Third, empirical evidence remains limited on the psychological mechanisms by which EI contributes to ELM in digital EFL contexts. While previous studies have reported positive relationships between EI and academic outcomes (Shengyao et al., 2024), comparatively little attention has been devoted to understanding the mediating role of AWB. This omission is important because emotionally intelligent learners may experience greater satisfaction, engagement, and emotional balance in their academic activities, which may, in turn, enhance their motivation to learn English. Therefore, the process through which EI translates into motivational outcomes remains insufficiently understood.

Fourth, although digital learning environments have become increasingly important in contemporary education, the role of perceived digital learning support (PDLS) remains underexplored. Existing studies have primarily focused on digital educational transformation and student engagement (Aldhafeeri and Alotaibi, 2022), while relatively little attention has been given to how EFL learners' perceptions of digital support influence the relationship between psychological AWB and ELM. Recent research suggests that supportive digital environments may enhance learner engagement and educational outcomes (Kok et al., 2025). However, the contextual conditions under which AWB contributes to ELM remain unclear. The global development of digital learning has also been uneven. While technologically advanced educational systems have increasingly integrated digital learning into instructional practice, developing contexts, including China, continue to face challenges related to infrastructure, pedagogical adaptation, and learner support (Ashraf et al., 2022). In China, the rapid expansion of online and blended learning has heightened concerns regarding learners' emotional engagement and sustained ELM, particularly in large EFL classrooms where individualized support may be limited (Li et al., 2026). These contextual realities highlight the importance of examining how emotional and environmental factors jointly influence motivational outcomes in digitally mediated language learning environments. To address these gaps, the present study develops and tests an integrated framework examining the relationships among EI, AWB, ELM, and PDLS in digital EFL settings. Specifically, the study investigates EI's direct and indirect effects on ELM through AWB. It examines PDLS's moderating influence. By integrating psychological and contextual perspectives within a single framework, the study contributes to a more comprehensive understanding of motivation in digital language learning environments. The findings provide theoretical insights into how emotional resources influence motivation and offer practical guidance for educators and policymakers seeking to enhance learner engagement and motivation in technology-enhanced EFL education. Based on the preceding discussion, the study pursues the following objectives:

1. To examine the effect of EI on AWB.
2. To investigate the relationship between EI and ELM.
3. To explore the effect of AWB on ELM.
4. To test the mediating role of AWB.
5. To examine the moderating role of PDLS.

Table 1 provides a structured synthesis of prior empirical and theoretical studies related to EI, AWB, ELM, and PDLS. By presenting these studies in a comparative format, the table clarifies how existing research has addressed these constructs across different educational contexts.

**Table 1:** Empirical evidence on EI, AWB, and ELM in EFL and PDLS contexts.

Authors	Context	Emotional Intelligence (EI)	Academic Well-being (AWB)	English Learning Motivation (ELM)	Perceived Digital Learning Support (PDLS)	Key focus	Research design
Villares et al. (2023)	General education	Yes	Yes	Yes	No	Emotional skills and academic outcomes	Quantitative
Llamas-Díaz et al. (2022)	Higher education	Yes	Yes	Limited	No	EI and well-being	Survey
Zhou et al. (2024)	Global education	Yes	Yes	Yes	No	EI and academic performance	Meta-analysis
Schnell et al. (2025)	School psychology	No	Yes	Yes	No	AWB and engagement	Longitudinal
Schukajlow et al. (2023)	Educational psychology	No	Yes	Yes	No	Emotions and motivation	Theoretical
Mahmoodi and Yousefi (2022)	EFL learning	Limited	No	Yes	No	Motivation in language learning	Conceptual
Guo et al. (2023)	Online learning	Limited	Yes	Yes	Yes	Digital learning support and engagement	Quantitative
Alenezi et al. (2023)	EFL in China	Limited	Yes	Yes	Limited	Digital learning challenges	Survey
Zhang et al. (2025)	Digital EFL	Limited	Limited	Yes	Yes	Online learning motivation	Mixed methods

Table 1 reveals both conceptual and methodological fragmentation in the existing literature. Conceptually, previous studies have examined different combinations of EI, AWB, ELM, and PDLS, but few have considered these four constructs within an integrated framework, particularly in digital EFL settings. Methodologically, the literature also varies considerably, including quantitative surveys, longitudinal research, meta-analyses, theoretical and conceptual studies, and mixed-methods designs. This diversity has generated valuable evidence from different perspectives. However, it has also limited direct comparison of how emotional, psychological, motivational, and digital-contextual factors operate simultaneously within a single explanatory model. Studies addressing digital learning have generally placed greater emphasis on engagement, technological conditions, or learning challenges. In contrast, studies examining EI and AWB have more commonly focused on psychological or academic outcomes. Consequently, evidence remains limited regarding the simultaneous direct, indirect, and conditional relationships among EI, AWB, ELM, and PDLS in digitally mediated EFL learning. The present study addresses this conceptual and methodological gap by examining these relationships within a unified structural model.

## 2 Literature review and hypotheses development

### 2.1 Theoretical foundations

This study is grounded in Self-Determination Theory (SDT) and Control-Value Theory (CVT), two influential perspectives that explain how psychological and contextual factors shape learning behavior and motivation. SDT proposes that motivation is strengthened when learners experience autonomy, competence, and relatedness within learning environments (Englund et al., 2023). In digital EFL settings, where learners often regulate their own learning activities, psychological resources become particularly important for maintaining engagement and persistence. Learners who possess stronger emotional competencies may be better equipped to cope with learning challenges and sustain motivation in technology-mediated environments. Control-Value Theory further explains how emotions influence academic engagement and achievement by emphasizing learners' perceptions of control and the value they assign to learning activities (Pekrun, 2024). According to this perspective, positive emotional experiences contribute to greater academic engagement and ELM, whereas negative emotional experiences may undermine learning outcomes. Consequently, emotional factors represent an important mechanism through which learners' internal resources affect their educational experiences. Within this theoretical framework, EI represents a key personal resource that enables learners to perceive, understand, regulate, and utilize emotions effectively (Antonopoulou, 2024). Learners with higher levels of EI are generally better able to manage academic stress, maintain positive emotional states, and respond constructively to learning difficulties. These capacities are especially important in digital learning environments, where learners often face challenges that require self-regulation and emotional adaptation.

AWB refers to learners' positive psychological functioning in educational settings, including feelings of engagement, satisfaction, emotional balance, and reduced academic stress (Huo, 2022). Drawing on SDT and CVT, emotionally intelligent learners are expected to experience higher levels of AWB because they can better regulate emotional responses and maintain positive learning experiences. Higher AWB, in turn, is expected to support stronger ELM by promoting sustained engagement and commitment to learning goals. ELM refers to the willingness, effort, and persistence that learners invest in acquiring English language skills (Wang et al., 2025a). As one of the most important predictors of language learning success, ELM influences how much learners engage in learning activities, overcome challenges, and continue pursuing language learning objectives over time. In digital EFL environments, maintaining ELM becomes particularly important because learners often have greater responsibility for directing and regulating their own learning processes. The study also incorporates PDLS as a contextual factor. SDT suggests that supportive learning environments strengthen learners' ELM by facilitating positive learning experiences and satisfying important psychological needs. Accordingly, learners who perceive greater levels of digital support may be more likely to translate positive psychological states into sustained ELM. PDLS, therefore, represents an important environmental condition that may strengthen the relationship between AWB and ELM. Drawing on SDT and CVT, the proposed framework suggests that EI contributes to ELM both directly and indirectly through AWB, while PDLS strengthens this relationship. Together, these theoretical perspectives provide a comprehensive foundation for understanding how emotional and contextual factors jointly influence ELM in digital EFL learning environments.

### 2.2 Global perspective on Emotional Intelligence (EI), Academic Well-being (AWB), and English Learning Motivation (ELM)

Recent developments in educational psychology have increasingly emphasized the importance of emotional and psychological factors in shaping learners' academic outcomes. Among these factors, EI has attracted considerable attention because it helps learners regulate emotions, manage stress, and adapt to challenging learning situations. Research across diverse educational contexts consistently shows that learners with higher EI tend to report greater engagement, better psychological adjustment, and more favorable academic outcomes (Zhoc et al., 2023). These findings suggest that emotional competencies represent important personal resources that support successful learning experiences. AWB has also emerged as a significant area of interest in educational research. AWB reflects learners' positive psychological functioning within academic environments and encompasses dimensions such as engagement, satisfaction, emotional balance, and reduced academic stress (Brandao De Souza and Jacomuzzi, 2025). Previous studies have shown that learners with higher levels of well-being are generally more engaged in educational activities and demonstrate greater persistence when facing academic challenges (Tang and Zhu, 2024). Consequently, AWB is an important factor contributing to both academic success and positive learning experiences. ELM remains one of the most extensively studied constructs in second language acquisition research. Motivation influences the effort, persistence, and commitment learners devote to language learning and has consistently been identified as a critical determinant of language learning success (Gao et al., 2024). Motivated learners are more likely to actively engage in learning activities, overcome difficulties, and maintain a long-term commitment to language acquisition. However, multiple psychological and contextual factors influence motivation, making it a complex and dynamic phenomenon. Despite substantial progress in these areas, existing research has often examined EI, AWB, and ELM independently. Although studies have established positive relationships among these constructs, they have paid limited attention to how they operate together within a unified framework, particularly in digital learning environments. As a result, important questions remain regarding the mechanisms through which emotional resources contribute to motivational outcomes and the contextual conditions that may strengthen or weaken these relationships. Addressing these issues is particularly important in contemporary EFL education, where digital technologies increasingly shape learning experiences and learner engagement.

### 2.3 EFL context

English as a Foreign Language (EFL) learning differs from many other educational domains because learners often have limited opportunities to use the target language outside formal educational settings. As a result, sustained ELM plays a particularly important role in determining language learning success. Previous research has consistently identified ELM as one of the strongest predictors of language learning achievement, influencing learners' effort, persistence, and long-term commitment to language acquisition (Wang and Xue, 2022). The rapid expansion of digital learning has further transformed the EFL learning experience. Online and blended learning environments provide greater flexibility and access to educational resources, but also require learners to assume greater responsibility for managing their own learning processes (Mulenga and Shilongo, 2025). In such environments, learners frequently encounter challenges related to self-regulation, reduced face-to-face interaction, and fluctuating engagement levels. These conditions increase the importance of psychological resources that help learners maintain ELM and adapt to changing learning demands. In China, digital and blended learning has developed rapidly in recent years. Although these developments have expanded educational access, they have also generated concerns regarding learner engagement, emotional adjustment, and sustained ELM, particularly in large EFL classes where individualized support may be limited (Jia and Cheng, 2022). Consequently, understanding how emotional and psychological factors influence ELM has become increasingly important within contemporary Chinese EFL education. Despite growing interest in psychological variables such as EI and AWB, existing EFL research has largely focused on instructional methods, classroom practices, and external motivational factors. Comparatively less attention has been devoted to understanding the psychological mechanisms through which emotional competencies contribute to ELM in digital learning environments. Furthermore, the potential role of PDLS as a contextual factor influencing motivational processes remains insufficiently explored. These limitations highlight the need for a more integrated perspective that considers both individual psychological resources and environmental conditions when examining ELM in digital EFL contexts.

### 2.4 Hypotheses development

#### 2.4.1 Emotional Intelligence (EI) and Academic Well-being (AWB)

EI is widely recognized as an important psychological resource that enables individuals to perceive, understand, regulate, and use emotions effectively (Quílez-Robres et al., 2023). In educational settings, emotionally intelligent learners are generally better equipped to manage academic stress, cope with learning difficulties, and maintain positive emotional states. These abilities support learners' psychological adjustment and overall well-being in academic environments. Previous studies have reported that learners with higher levels of EI tend to experience greater academic satisfaction, engagement, and psychological resilience (Nǎstasǎ et al., 2022). In digital learning environments, where learners often face increased autonomy and reduced direct support, emotional regulation may become even more important for maintaining positive educational experiences. Consequently, emotionally intelligent learners are expected to demonstrate higher levels of AWB.

**H1:** Emotional intelligence (EI) positively influences academic well-being (AWB).

#### 2.4.2 Emotional Intelligence (EI) and English Learning Motivation (ELM)

EI is also expected to play a significant role in shaping ELM. According to Self-Determination Theory, learners who effectively regulate their emotions are better able to sustain effort, maintain engagement, and persist in pursuing learning goals. Language learning often involves challenges such as communication anxiety, fear of making mistakes, and frustration associated with skill development. Learners with stronger emotional competencies are more likely to manage these challenges effectively and remain committed to language learning activities. Previous research has demonstrated positive relationships between EI and motivational outcomes across various educational contexts (Arias et al., 2022). In digital EFL environments, where learners often rely on self-directed learning and independent engagement, EI may be a particularly important source of ELM.

**H2:** Emotional intelligence (EI) positively influences English learning motivation (ELM).

#### 2.4.3 Academic Well-being (AWB) and English Learning Motivation (ELM)

AWB reflects learners' positive psychological functioning within educational settings, including satisfaction, engagement, emotional balance, and reduced academic stress (Wang et al., 2025b). Learners with higher well-being are generally more likely to maintain positive attitudes toward learning, invest effort in academic activities, and remain committed to educational goals. Prior research has shown that AWB contributes to learner engagement, persistence, and educational success (Huo, 2022). In digital EFL contexts, where ELM may fluctuate due to technological and environmental challenges, positive academic experiences can play an important role in sustaining learners' commitment to language learning. Therefore, learners who report higher levels of AWB are expected to demonstrate stronger ELM.

**H3:** Academic well-being (AWB) positively influences English learning motivation (ELM).

#### 2.4.4 Mediating Role of Academic Well-being (AWB)

AWB is expected to mediate the relationship between EI and ELM. EI contributes to learners' ability to manage emotions and maintain positive psychological functioning, thereby enhancing AWB. Higher levels of AWB may subsequently encourage greater engagement, persistence, and motivation toward language learning activities. Previous studies have suggested that psychological well-being frequently serves as an intermediate mechanism through which personal resources influence behavioral and motivational outcomes (Kubzansky et al., 2023). From this perspective, AWB offers a theoretical explanation of how emotional competencies translate into motivational outcomes in educational settings.

**H4:** Academic well-being (AWB) mediates the relationship between emotional intelligence (EI) and English learning motivation (ELM).

#### 2.4.5 Moderating Role of Perceived Digital Learning Support (PDLS)

PDLS refers to learners' perceptions of the resources, guidance, feedback, and assistance available within digital learning environments. Supportive digital environments can facilitate learner engagement by providing conditions that enable students to use their psychological resources effectively. Previous research has emphasized the importance of digital support in promoting engagement, satisfaction, and educational outcomes in online learning environments (Singh and Ishrat, 2025). Learners who perceive higher levels of digital support may be better positioned to convert positive psychological experiences into sustained motivation. Consequently, PDLS is expected to strengthen the positive relationship between AWB and ELM. In other words, the motivational benefits associated with AWB are likely to be greater when learners perceive stronger PDLS.

**H5:** Perceived digital learning support (PDLS) moderates the relationship between academic well-being (AWB) and English learning motivation (ELM), such that the relationship is stronger when PDLS is higher.

### 2.5 Research gap and conceptual framework

Although previous studies have established the importance of EI, AWB and ELM, important theoretical and contextual gaps remain. Existing research has predominantly examined these variables in isolation or through simple direct relationships, providing a limited understanding of how emotional resources are transformed into motivational outcomes within digitally mediated EFL environments (Wei, 2022). As a result, the psychological mechanisms by which EI influences ELM remain insufficiently explained. Furthermore, the rapid expansion of digital learning has altered the conditions under which language learning occurs. Learners are increasingly required to regulate their own learning activities, manage emotional challenges, and maintain motivation within technology-supported environments (Stalmach et al., 2025). While prior studies have highlighted the importance of PDLS and learner engagement, relatively little attention has been given to its role as a contextual condition that may strengthen or weaken the impact of psychological factors on motivational outcomes (Bos-Nehles et al., 2023). To address these limitations, the present study develops an integrated framework that combines emotional, psychological, and contextual perspectives. EI is positioned as a personal resource that directly enhances ELM and indirectly influences it through AWB. AWB is conceptualized as a mediating mechanism because it reflects learners' psychological functioning in educational settings and explains how emotional competencies translate into motivational outcomes. PDLS is included as a moderating factor because supportive digital environments may enhance the extent to which positive psychological states contribute to sustained motivation for learning. By integrating these relationships into a single framework, the study extends existing research on digital EFL learning. It provides a more comprehensive explanation of how individual and contextual factors jointly influence motivation to learn English. The proposed framework offers theoretical and practical insights into how emotional resources contribute to successful language learning in technology-enhanced educational environments.

The proposed framework positions EI as a key psychological resource that influences ELM directly and indirectly through AWB. AWB explains how EI contributes to ELM, while PDLS shapes the strength of this relationship. This framework coherently explains how psychological and contextual factors interact within digital EFL learning environments. Figure 1 presents the study's conceptual framework and illustrates the hypothesized relationships among EI, AWB, ELM, and PDLS.

**Figure 1:**
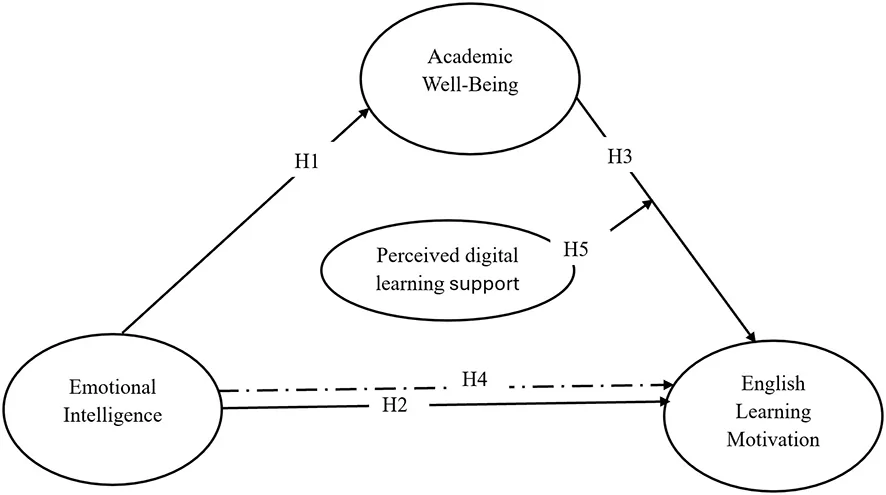
Conceptual framework of the study, showing the various hypotheses. Author's own work.

The figure clarifies the proposed model's structure by showing the direct, mediating, and moderating relationships. It clearly shows how the theoretical constructs are linked and supports the transition from conceptual development to empirical analysis.

## 3 Methodology

### 3.1 Research design and analytical approach

The study adopted a quantitative, cross-sectional design to examine the relationships among EI, AWB, ELM, and PDLS in English as a Foreign Language contexts. This design was appropriate because the research examined theoretically derived direct, indirect, and moderating associations within a structured conceptual framework. We conducted partial least squares structural equation modeling using WarpPLS 8.0. This approach suits models with multiple latent constructs, indirect associations, and interaction terms without imposing strict multivariate normality assumptions (Wang et al., 2024). We examined the indirect association between EI and ELM through AWB using indirect-effect analysis. The moderating role of PDLS was evaluated through the interaction between AWB and PDLS. The available analysis employed the Stable3 resampling procedure with 100 resamples to estimate standard errors and probability values. Table 2 presents the construct specifications and indicator codes used in the revised WarpPLS analysis.

**Table 2:** Study constructs and measurement items.

Construct	Analytical item codes	Number of indicators	Measurement focus	Specification	Response scale
Emotional Intelligence (EI)	EI1–EI5	5	Learners' capacity to recognize, understand, regulate, and use emotions during English learning	Reflective	Seven-point Likert scale
Academic Well-being (AWB)	AWB1–AWB5	5	Learners' satisfaction, emotional balance, engagement, and psychological functioning in the academic environment	Reflective	Seven-point Likert scale
English Learning Motivation (ELM)	ELM1–ELM5	5	Learners' willingness to invest effort, persist during difficulties, and remain committed to improving English proficiency	Reflective	Seven-point Likert scale
Perceived Digital Learning Support (PDLS)	PDLS1–PDLS5	5	Learners' perceptions of the resources, feedback, guidance, accessibility, and assistance available in digital learning environments	Reflective	Seven-point Likert scale

The table demonstrates that all items were adapted from established scales and aligned with the digital EFL context. This supports the instrument's content validity and ensures transparency in the measurement process.

### 3.2 Sampling strategy and data collection

The target population comprised college and university students studying English as a Foreign Language (EFL) in Henan Province, China. The study used a convenience sampling strategy because recruitment depended on institutional accessibility, participant availability, and willingness to participate. Convenience sampling is appropriate when eligible participants are recruited based on accessibility; however, its non-probability nature limits population-level representativeness and the generalizability of the findings beyond the sampled institutions (Alaba et al., 2026). Data were collected between September and December 2025 from participating educational institutions in Henan Province. Participants were eligible if they were at least 18 years old, actively enrolled in an EFL program, experienced in digitally supported English-learning activities, and willing to provide written informed consent. Respondents were excluded if they were younger than 18 years, did not provide informed consent, failed to meet the EFL or digital-learning eligibility requirements, or submitted questionnaires that did not satisfy the established data-quality screening criteria. Participant recruitment and screening were conducted sequentially during the designated data-collection period. The initial dataset contained 435 responses. A subsequent respondent-level eligibility audit identified 52 participants who were younger than 18 years. Because the approved consent procedure applied exclusively to adult participants, all 52 responses were permanently excluded before the revised analysis, leaving 383 eligible adult respondents. Recruitment subsequently continued using the same participating institutions, questionnaire, eligibility requirements, and data-collection procedures. Fifty-two additional respondents aged 18 years or older were recruited and screened, resulting in a final adult-only analytical sample of 435 participants. None of the original responses submitted by participants younger than 18 years was retained in the revised dataset. All demographic, measurement-model, structural-model, mediation, moderation, and model-quality analyses reported in the manuscript were conducted using the final dataset of 435 eligible adult respondents.

Sample adequacy was evaluated using the inverse square root and gamma-exponential methods developed specifically for minimum sample-size estimation in PLS-SEM (Ali, 2026). Based on a significance level of 0.05, statistical power of 0.80, and a conservative minimum absolute path coefficient of 0.130, the inverse square root method indicated a minimum required sample of 366 respondents, whereas the gamma-exponential method indicated a minimum of 353 respondents. The final adult-only sample of 435 exceeded both requirements and was therefore considered adequate for estimating the proposed PLS-SEM model. Before questionnaire completion, each participant received an information sheet explaining the study's purpose, procedures, voluntary nature, intended use of the collected data, and measures adopted to protect anonymity and confidentiality. Written informed consent was obtained before participation. Participants were informed that they could decline participation or withdraw without penalty. Participants completed the questionnaires anonymously, and the study collected no personally identifiable or sensitive personal information. College and university students were pooled because both groups possessed the characteristics central to the research model: active participation in EFL learning and experience with digitally supported English-learning activities. Their inclusion enabled the relationships among EI, AWB, ELM, and PDLS to be examined across a broader digitally supported EFL context. Nevertheless, pooling the two educational groups does not imply that they are educationally, developmentally, or psychometrically equivalent. College and university students may differ in their academic demands, learning autonomy, developmental characteristics, English-learning experience, and exposure to digital learning technologies. Such differences could influence both the measurement properties of the constructs and the estimated structural relationships.

The study did not conduct a formal Measurement Invariance of Composite Models (MICOM) assessment or multigroup analysis. The structural coefficients are therefore interpreted as overall associations within the pooled adult sample and not as evidence that the constructs were measured equivalently or that the structural relationships were identical across college and university students. No conclusions are drawn regarding differences in individual path coefficients between the two educational groups. Future studies should apply the MICOM procedure to evaluate configural invariance, compositional invariance, and the equality of composite means and variances before undertaking group comparisons (Beltrão et al., 2025). Once the required level of measurement invariance has been established, PLS multigroup analysis can be used to determine whether specific structural relationships differ significantly across educational groups (Omanyo et al., 2026).

### 3.3 Instrument development and validation

The study employed a structured questionnaire to measure EI, AWB, ELM, and PDLS. The measurement items were adapted from previously published instruments and contextually modified for digitally supported English as a Foreign Language learning. The modifications were limited to contextual wording and did not intentionally alter the conceptual domain represented by each original measure. EI captured learners' capacity to recognize, understand, regulate, and use emotions during English-learning activities. AWB represented satisfaction, emotional balance, engagement, and constructive responses to academic demands. ELM reflected learners' willingness to invest effort, persist when learning became difficult, and remain committed to improving English proficiency. PDLS captured learners' perceptions of the resources, feedback, guidance, accessibility, and assistance available in digital learning environments. The initial questionnaire was evaluated by a panel of five academic experts. Three experts specialized in English language education and applied linguistics, while two specialized in educational psychology and quantitative research methods. Each expert independently assessed the conceptual relevance, clarity, contextual appropriateness, and construct alignment of the items. The experts were also requested to identify ambiguous wording and potential conceptual overlap among EI, AWB, ELM, and PDLS. Their evaluations resulted in minor linguistic and contextual revisions without changing the intended conceptual meaning of the constructs. Because the collected data were from EFL learners in China, we prepared the questionnaire in Chinese using a forward-translation and back-translation procedure. Two bilingual researchers independently translated the original English items into Chinese. The team compared and reconciled the translations to produce a preliminary Chinese questionnaire. A different bilingual specialist, who had not examined the original items during the initial translation stage, translated the reconciled Chinese version back into English. The research team compared the back-translated version with the original questionnaire to identify differences in meaning, terminology, and contextual interpretation. The team discussed and corrected any discrepancies before the pilot assessment. The team then conducted a pilot assessment with 30 EFL learners who met the main study's eligibility criteria but were not included in the final analytical sample. Participants evaluated item comprehensibility, instruction clarity, response-scale suitability, and the time required to complete the questionnaire. Participant feedback was used to revise unclear expressions and improve the questionnaire's contextual relevance. We did not add, remove, or alter any construct or hypothesized relationship after the expert review or pilot assessment. The pilot procedure aimed to evaluate questionnaire clarity and administration feasibility rather than provide definitive evidence of measurement reliability or validity. We then reassessed measurement quality using the final analytical sample, and report the resulting indicator reliability, internal consistency reliability, convergent validity, and discriminant validity statistics in the Results section.

The study assessed the measurement properties of all constructs using WarpPLS 8.0. EI, AWB, ELM, and PDLS were specified as reflective constructs because their indicators were conceptualized as manifestations of the corresponding latent variables. Indicator reliability was evaluated using outer loadings, while internal consistency reliability was examined using Cronbach's alpha and composite reliability. Cronbach's alpha values were 0.887 for EI, 0.899 for AWB, 0.892 for ELM, and 0.906 for PDLS. The corresponding composite reliability values were 0.917, 0.925, 0.920, and 0.930. Convergent validity was evaluated using the average variance extracted, which produced values of 0.689, 0.712, 0.698, and 0.727, respectively. These results exceeded the recommended thresholds of 0.70 for internal consistency reliability and 0.50 for convergent validity. Discriminant validity was assessed using the heterotrait-monotrait ratio, with all construct-pair values remaining below the conservative threshold of 0.85. Therefore, the revised measurement model demonstrated satisfactory indicator reliability, internal consistency reliability, convergent validity, and discriminant validity.

### 3.4 Measurement scales

All measurement items were evaluated using a seven-point Likert scale ranging from 1 (strongly disagree) to 7 (strongly agree). The same response format was applied consistently across EI, AWB, ELM, and PDLS. A seven-point scale was used to capture greater variation in learners' responses while maintaining a uniform and comprehensible response structure throughout the questionnaire. This specification matches the response scale reported for all four constructs in Table 2.

### 3.5 Data quality and bias control

Given the single-source, cross-sectional survey design, the authors addressed potential common method bias through procedural and statistical measures. Procedurally, respondent anonymity was protected, questionnaire wording was designed to minimize response bias, and the construct sections were separated to reduce cognitive carryover between measures. Statistically, we assessed full collinearity using variance inflation factors in WarpPLS 8.0. The values were 1.289 for EI, 1.204 for AWB, 1.353 for ELM, and 1.064 for PDLS. All values were substantially below the recommended threshold of 3.3, indicating that full collinearity and common method bias were unlikely to represent substantial concerns. Indicator-level collinearity was also assessed, with variance inflation factor values ranging from 1.779 to 2.821 and therefore remaining within acceptable limits.

### 3.6 Mediation and moderation analysis

Mediation was examined by estimating the indirect association between EI and ELM through AWB. Moderation was assessed using the interaction between AWB and PDLS. The available WarpPLS analysis employed the Stable3 resampling procedure with 100 resamples to estimate standard errors and probability values. Accordingly, the indirect and interaction associations were evaluated using the coefficients, standard errors, and probability values generated by this procedure. The simple slopes and conditional indirect-effect values were derived algebraically from the standardized coefficients and were used only to describe the observed interaction pattern. Because conventional bootstrapped confidence intervals were unavailable, these derived values were not treated as inferentially tested conditional effects. The mediation and moderation findings are therefore interpreted as provisional statistical associations requiring confirmation through conventional bootstrapping with a larger number of resamples.

### 3.7 Model validation

Model quality was evaluated using the statistical indices generated by WarpPLS 8.0. Indicator-level and full-collinearity variance inflation factors were examined to assess collinearity. The coefficient of determination and adjusted coefficient of determination were used to evaluate the explanatory power of the endogenous constructs, while the Stone-Geisser predictive-relevance coefficient was used to assess predictive relevance. Overall model quality was also evaluated using the average path coefficient, average coefficient of determination, average adjusted coefficient of determination, average block variance inflation factor, average full-collinearity variance inflation factor, Tenenhaus goodness-of-fit index, Simpson's paradox ratio, coefficient-of-determination contribution ratio, statistical suppression ratio, and non-linear bivariate causality-direction ratio. Statistical significance was estimated using the Stable3 procedure with 100 resamples. Because conventional bootstrap confidence intervals were unavailable, conclusions concerning the indirect and interaction associations were interpreted cautiously.

## 4 Results

### 4.1 Descriptive results of the participants

The sample in this study consists of EFL learners from different educational levels and learning backgrounds. Participants were drawn from college and university contexts, indicating that the dataset captures learners at different stages of formal education. This diversity is consistent with prior research, which emphasizes that learner characteristics vary across educational levels and may influence ELM (Anyichie and Butler, 2023). The sample also reflects variation in English and digital learning experiences. This variation suggests differences in learners' exposure to language learning and familiarity with digital environments. Previous studies have shown that such differences can affect both engagement and perceptions of digital support (Aleti et al., 2025). The study employed a quantitative survey design, and no interview data were collected. Therefore, the results presented in this section are based solely on the statistical analysis conducted using WarpPLS. Table 3 provides an overview of respondents' demographic characteristics. It summarizes key attributes such as gender, age, educational level, and learning experience.

**Table 3:** Respondents' demographic characteristics (*n* = 435).

Variable	Category	Estimated frequency	Percentage (%)
Gender	Male	201	46.2
	Female	224	51.5
	Prefer not to say	10	2.3
	**Total**	**435**	**100.0**
Age	18–22 years	310	71.3
	23–30 years	92	21.1
	Above 30 years	33	7.6
	**Total**	**435**	**100.0**
Educational level	College	315	72.4
	University	120	27.6
	**Total**	**435**	**100.0**
English-learning experience	Less than 3 years	101	23.2
	3–5 years	139	32.0
	6–10 years	130	29.9
	More than 10 years	65	14.9
	**Total**	**435**	**100.0**
Digital-learning experience	Low	119	27.4
	Moderate	207	47.6
	High	109	25.1
	**Total**	**435**	**100.1**

Bold entries indicate the demographic variables and the corresponding totals for each variable. Percentages may not sum to exactly 100.0% because of rounding.

The table shows that the sample includes learners from diverse educational backgrounds and varying levels of experience. This diversity strengthens the findings' relevance by reflecting a range of learner conditions in digital EFL contexts.

### 4.2 Model diagnostics and structural model results

Following assessment of the measurement model, the structural associations were examined using WarpPLS 8.0. The results are shown in Figure 2. The model fit and quality indices indicated acceptable overall performance. The average path coefficient was 0.273 (*p* < 0.001), the average coefficient of determination was 0.184 (*p* < 0.001), and the average adjusted coefficient of determination was 0.181 (*p* < 0.001). The average block variance inflation factor was 1.100, while the average full collinearity variance inflation factor was 1.191. Both values were substantially below the recommended threshold of 3.3. The Tenenhaus goodness of fit index was 0.375, while the Simpson's paradox ratio, coefficient of determination contribution ratio, statistical suppression ratio, and non-linear bivariate causality direction ratio were all 1.000. EI was positively associated with AWB (β = 0.354, *p* < 0.001), supporting H1. EI was also positively associated with ELM (β = 0.331, *p* < 0.001), supporting H2. AWB was positively associated with ELM (β = 0.229, *p* < 0.001), supporting H3. The Stable3 output additionally indicated a positive specific indirect association between EI and ELM through AWB (β = 0.081, *SE* = 0.034, *p* = 0.008, *f*^2^ = 0.033). It also indicated a positive interaction between AWB and PDLS in relation to ELM (β = 0.180, *SE* = 0.047, *p* < 0.001, *f*^2^ = 0.029). These findings provided provisional support for H4 and H5. Because the analysis employed 100 Stable3 resamples and did not generate conventional bootstrapped confidence intervals, the indirect and interaction results should not be regarded as inferential confirmation of mediation or moderation. The model explained 12.5% of the variance in AWB and 24.3% of the variance in ELM. The corresponding adjusted coefficients of determination were 0.123 and 0.238, while the Stone-Geisser predictive relevance coefficients were 0.126 and 0.244. Because the research employed a cross-sectional design, all structural coefficients are interpreted as statistical associations rather than evidence of causal or temporal relationships.

**Figure 2:**
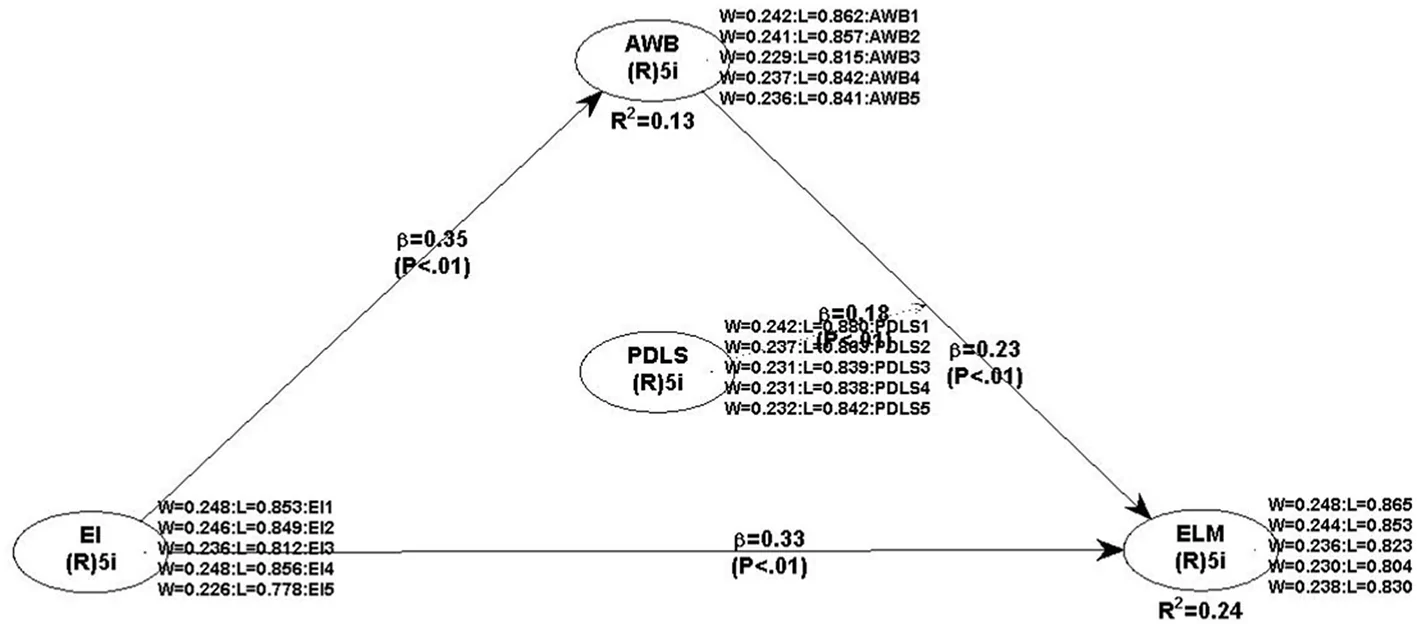
Original WarpPLS graphical output for the structural model. Author's own work. EI, Emotional Intelligence; AWB, Academic Well-being; ELM, English Learning Motivation; PDLS, Perceived Digital Learning Support. The figure presents the revised WarpPLS structural model based on 435 usable responses. EI was positively associated with AWB (β = 0.354, *p* < 0.001) and ELM (β = 0.331, *p* < 0.001). AWB was positively associated with ELM (β = 0.229, *p* < 0.001), while PDLS positively moderated the relationship between AWB and ELM (β = 0.180, *p* < 0.001). The model explained 12.5% of the variance in AWB and 24.3% of the variance in ELM. Values displayed graphically may be rounded to two decimal places; Table 7 reports the precise coefficients and probability values.

EI was positively associated with AWB (β = 0.354, *p* < 0.001), supporting H1. EI was also positively associated with ELM (β = 0.331, *p* < 0.001), supporting H2. AWB was positively associated with ELM (β = 0.229, *p* < 0.001), supporting H3. The Stable3 output indicated a positive specific indirect association between EI and ELM through AWB (β = 0.081, *p* = 0.008), providing provisional support for H4. It also indicated a positive interaction between AWB and PDLS in relation to ELM (β = 0.180, *p* < 0.001), providing provisional support for H5. Because the analysis employed 100 Stable3 resamples without conventional bootstrapped confidence intervals, H4 and H5 should not be interpreted as inferentially confirmed mediation and moderation effects. The explanatory and predictive power of the model was subsequently evaluated using the coefficient of determination and the Stone-Geisser predictive relevance coefficient. The explanatory and predictive power of the model was subsequently evaluated using the coefficient of determination and the Stone-Geisser predictive-relevance coefficient:


$$\begin{array}{c} R^{2}=1-\frac{\sum {\left(y_{i}-{ŷ}_{i}\right)}^{2}}{\sum {\left(y_{i}-\overset{-}{y}\right)}^{2}} \end{array}$$

The model explained 12.5% of the variance in AWB (*R*^2^ = 0.125) and 24.3% of the variance in ELM (*R*^2^ = 0.243). The corresponding adjusted coefficients of determination were 0.123 and 0.238, respectively. These results indicate modest explanatory power for AWB and moderate explanatory power for ELM. The Stone-Geisser predictive-relevance coefficients were 0.126 for AWB and 0.244 for ELM. Because both values exceeded zero, the model demonstrated acceptable predictive relevance for the two endogenous constructs. Nevertheless, a substantial proportion of the variance remained unexplained, indicating that English-learning outcomes may also be associated with psychological, instructional, social, and contextual factors not included in the present model. The moderation relationship was estimated using the interaction between AWB and PDLS and can be expressed as follows:


$$\begin{array}{c} ELM=\beta_{0}+\beta_{1}\left(AWB\right)+\beta_{2}\left(PDLS\right)+\beta_{3}\left(AWB\times PDLS\right)+\varepsilon \end{array}$$

The positive interaction coefficient (β = 0.180, *p* < 0.001) indicates that the association between AWB and ELM became stronger as PDLS increased. The descriptive standardized simple slope was approximately 0.049 when PDLS was one standard deviation below its mean, 0.229 at its mean, and 0.409 when it was one standard deviation above its mean. Figures 3, 4 illustrate this descriptive interaction pattern across lower and higher levels of PDLS. Because the analysis employed 100 Stable3 resamples without conventional bootstrapped confidence intervals, the interaction pattern provides provisional support for H5 and should not be regarded as inferential confirmation of moderation.

**Figure 3:**
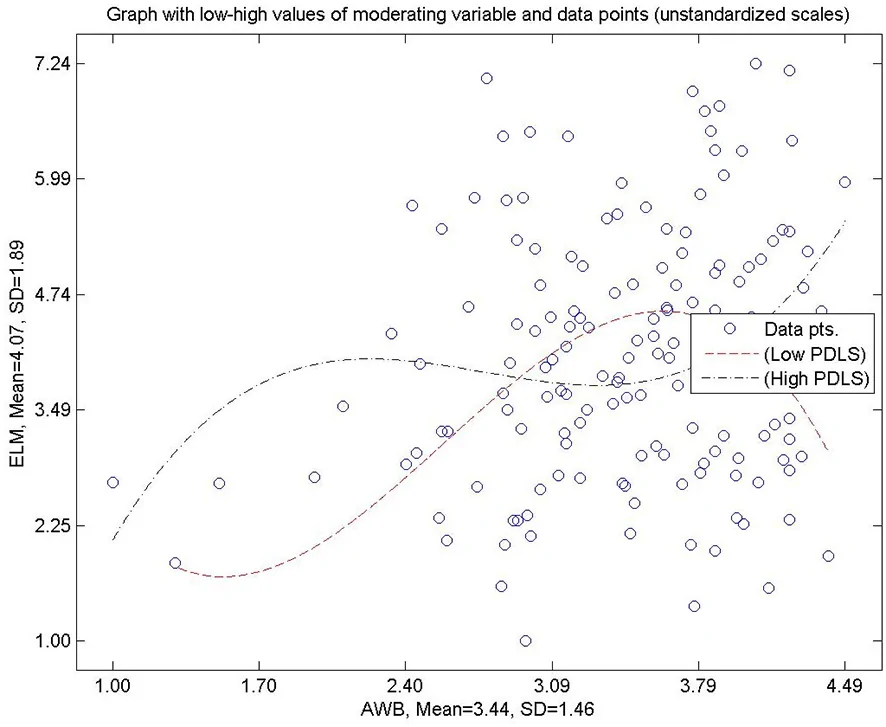
Non-linear graph displaying the impact of AWB on ELM across low and high degrees of PDLS. Author's own work.

**Figure 4:**
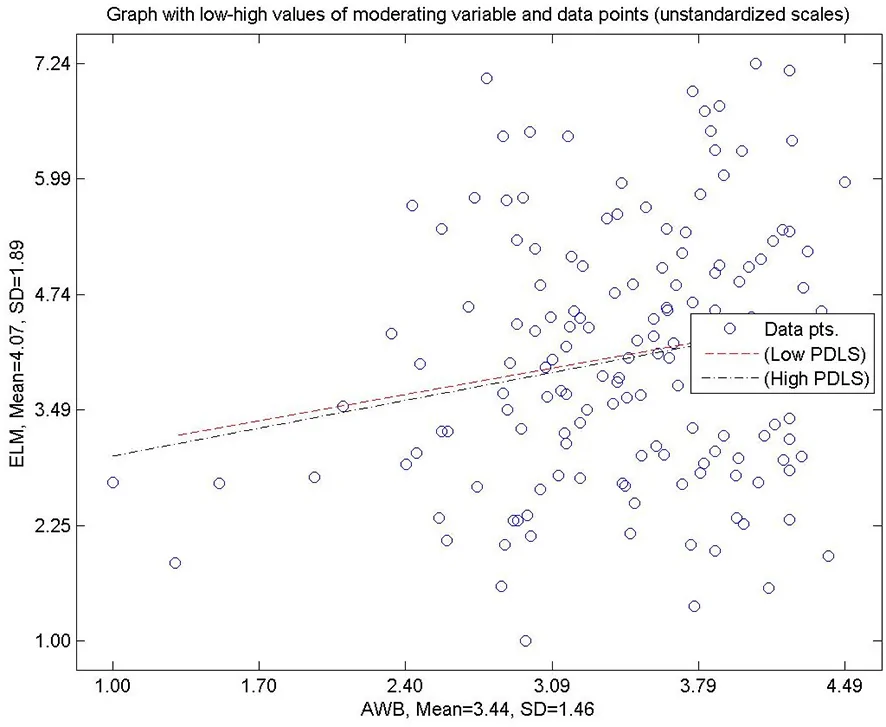
Linear graph displaying the impact of AWB on ELM across low and high degrees of PDLS. Author's own work.

Figure 4 presents a linear interaction plot that shows the relationship between AWB and ELM at different levels of PDLS. The figure indicates that the slope of the relationship becomes steeper at higher PDLS. This supports the interpretation that digital support strengthens AWB's influence on ELM.

Figure 5 provides a descriptive three-dimensional representation of the interaction involving AWB, PDLS, and ELM. The surface illustrates how the estimated association between AWB and ELM varies across levels of PDLS. Because conventional bootstrapped confidence intervals were unavailable, the figure should be interpreted as a visual description of the observed interaction pattern rather than inferential confirmation of moderation.

**Figure 5:**
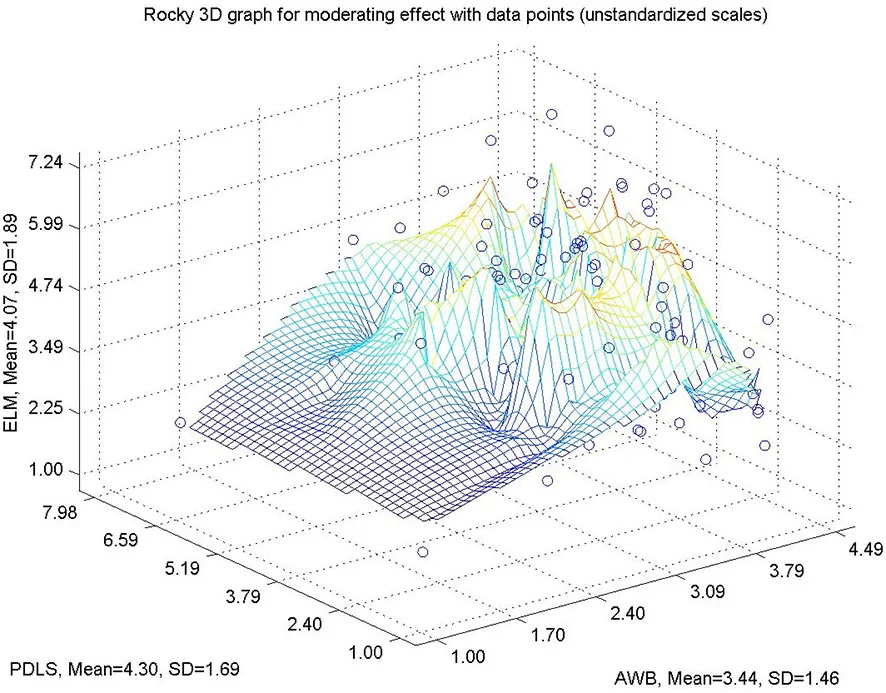
Three-dimensional interaction surface. Author's own work.

The measurement properties of EI, AWB, ELM, and PDLS were assessed according to their reflective specifications. The reflective specification was retained because the observed indicators were conceptualized as manifestations of their corresponding latent constructs rather than as components that collectively formed them. The assessment therefore examined indicator reliability, internal consistency reliability, convergent validity, discriminant validity, and indicator-level collinearity. Indicator reliability was evaluated using outer loadings. Cronbach's alpha and composite reliability were used to assess internal consistency reliability, while average variance extracted was used to assess convergent validity. Discriminant validity was evaluated using the heterotrait-monotrait ratio, and indicator-level variance inflation factors were examined to identify potential collinearity among the measurement items. Table 4 presents the indicator loadings, *p*-values, and variance inflation factor values obtained from the revised WarpPLS analysis. All retained indicators loaded strongly and significantly on their corresponding constructs. The loadings ranged from 0.778 to 0.856 for EI, from 0.815 to 0.862 for AWB, from 0.804 to 0.865 for ELM, and from 0.838 to 0.880 for PDLS. All indicator loadings exceeded 0.70 and were statistically significant at *p* < 0.001, establishing satisfactory indicator reliability. Indicator-level variance inflation factor values ranged from 1.779 to 2.821. All values remained below the general threshold of 5.0, indicating that indicator-level collinearity was not a substantial concern.

**Table 4:** Measurement model assessment.

Construct	Indicator	Outer loading	*p*-value	VIF
Emotional Intelligence (EI)	EI1	0.853	< 0.001	2.378
EI	EI2	0.849	< 0.001	2.330
EI	EI3	0.812	< 0.001	1.990
EI	EI4	0.856	< 0.001	2.390
EI	EI5	0.778	< 0.001	1.779
Academic Well-being (AWB)	AWB1	0.862	< 0.001	2.508
AWB	AWB2	0.857	< 0.001	2.457
AWB	AWB3	0.815	< 0.001	2.049
AWB	AWB4	0.842	< 0.001	2.281
AWB	AWB5	0.841	< 0.001	2.298
English Learning Motivation (ELM)	ELM1	0.865	< 0.001	2.551
ELM	ELM2	0.853	< 0.001	2.389
ELM	ELM3	0.823	< 0.001	2.081
ELM	ELM4	0.804	< 0.001	1.934
ELM	ELM5	0.830	< 0.001	2.155
Perceived Digital Learning Support (PDLS)	PDLS1	0.880	< 0.001	2.821
PDLS	PDLS2	0.863	< 0.001	2.577
PDLS	PDLS3	0.839	< 0.001	2.273
PDLS	PDLS4	0.838	< 0.001	2.261
PDLS	PDLS5	0.842	< 0.001	2.299

Author's own work.

These results above support the construct-level reliability and convergent-validity evidence reported in Table 5. As reported in Table 5, Cronbach's alpha coefficients ranged from 0.887 to 0.906, composite reliability values ranged from 0.917 to 0.930, and average variance extracted values ranged from 0.689 to 0.727. These results establish satisfactory indicator reliability, internal consistency reliability, and convergent validity for EI, AWB, ELM, and PDLS.

**Table 5:** Internal consistency reliability and convergent validity.

Construct	Indicators	Loading range	Cronbach's alpha	Composite reliability	Average variance extracted	Full collinearity VIF
Emotional Intelligence (EI)	5	0.778–0.856	0.887	0.917	0.689	1.289
Academic Well-being (AWB)	5	0.815–0.862	0.899	0.925	0.712	1.204
English Learning Motivation (ELM)	5	0.804–0.865	0.892	0.920	0.698	1.353
Perceived Digital Learning Support (PDLS)	5	0.838–0.880	0.906	0.930	0.727	1.064

Author's own work.

As shown in Figure 6, the minimum required sample size was estimated using a conservative minimum absolute significant path coefficient of 0.130, a significance level of 0.05, and statistical power of 0.80. The inverse square root method indicated a minimum required sample size of 366, while the gamma-exponential method indicated a minimum of 353. The final analytical sample comprised 435 respondents and therefore exceeded both minimum sample-size requirements. Accordingly, the achieved sample size was considered adequate for estimating the proposed partial least squares structural equation model.

**Figure 6:**
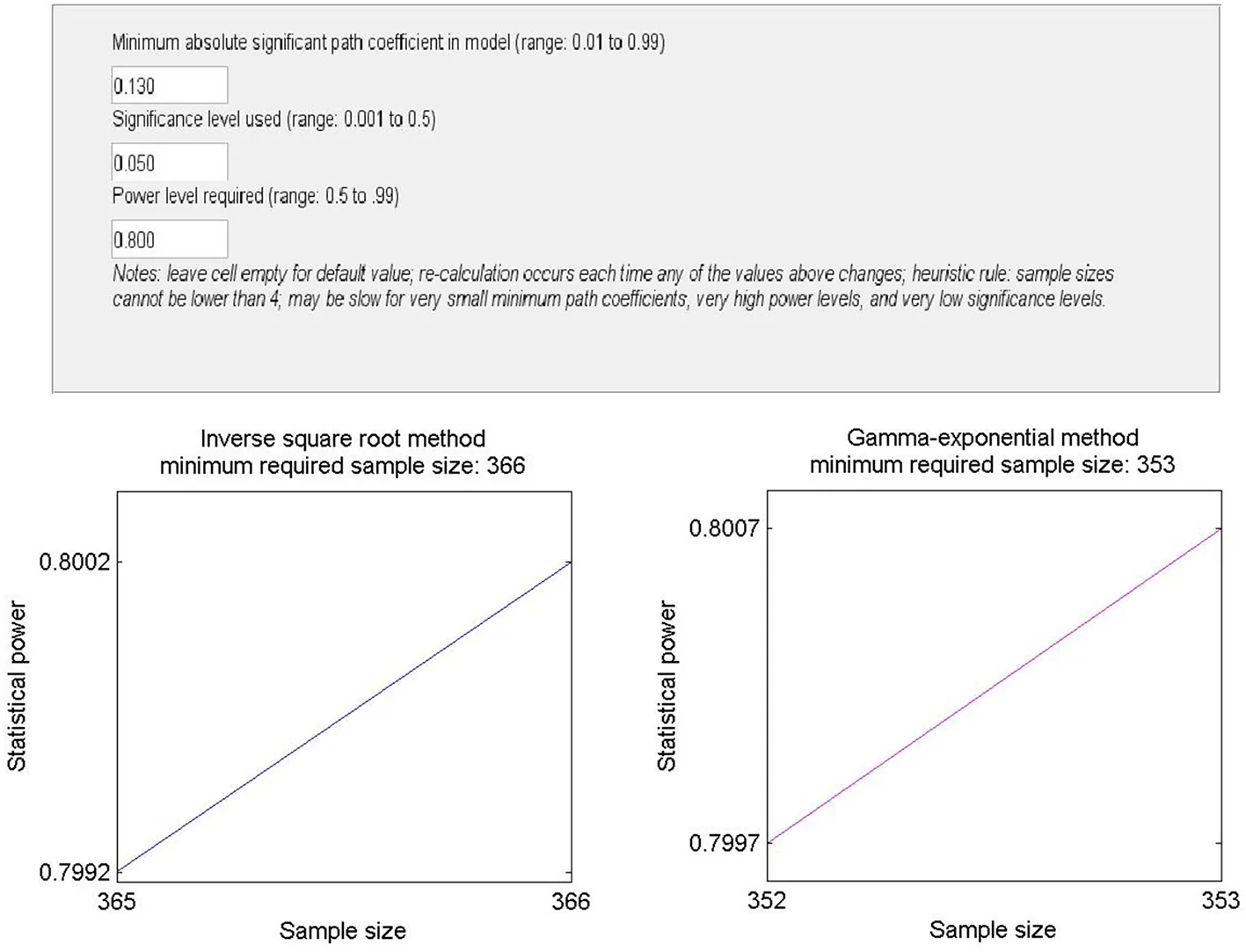
Minimum required sample size based on the inverse square root and gamma-exponential methods.

### 4.3 Reliability, convergent validity, and discriminant validity

The reflective measurement model was evaluated through indicator reliability, internal consistency reliability, convergent validity, and discriminant validity in Tables 5, 6. All retained indicators demonstrated strong and statistically significant loadings on their corresponding constructs. The indicator loadings ranged from 0.778 to 0.856 for EI, from 0.815 to 0.862 for AWB, from 0.804 to 0.865 for ELM, and from 0.838 to 0.880 for PDLS. All loadings were statistically significant at *p* < 0.001 and exceeded the recommended minimum threshold. These results provide satisfactory evidence of indicator reliability. Internal consistency reliability was established for all four constructs. Cronbach's alpha coefficients were 0.887 for EI, 0.899 for AWB, 0.892 for ELM, and 0.906 for PDLS. Composite reliability values were 0.917, 0.925, 0.920, and 0.930, respectively. All values exceeded the recommended threshold of 0.70 and remained below 0.95, demonstrating satisfactory internal consistency without indicating excessive indicator redundancy. Convergent validity was assessed using the average variance extracted. The values were 0.689 for EI, 0.712 for AWB, 0.698 for ELM, and 0.727 for PDLS. Each value exceeded the recommended threshold of 0.50, indicating that the indicators explained more than half of the variance in their corresponding constructs. Discriminant validity was evaluated using the heterotrait-monotrait ratio of correlations. The ratios were 0.386 between EI and AWB, 0.449 between EI and ELM, 0.184 between EI and PDLS, 0.379 between AWB and ELM, 0.125 between AWB and PDLS, and 0.258 between ELM and PDLS. All values were below the conservative threshold of 0.85. The heterotrait-monotrait ratio between EI and PDLS was 0.184, which was substantially below the conservative threshold of 0.85. This result confirms that EI and PDLS were empirically distinguishable in the revised measurement model. Therefore, EI and PDLS were empirically distinguishable in the revised analysis. Full collinearity variance inflation factor values were 1.289 for EI, 1.204 for AWB, 1.353 for ELM, and 1.064 for PDLS. All values were substantially below 3.3, indicating that full collinearity was not a substantial concern. Overall, the revised measurement model established satisfactory indicator reliability, internal consistency reliability, convergent validity, and discriminant validity.

**Table 6:** HTMT Assessment of discriminant validity.

Construct pair	HTMT	Criterion	Assessment
EI–AWB	0.386	< 0.85	Established
EI–ELM	0.449	< 0.85	Established
EI–PDLS	0.184	< 0.85	Established
AWB–ELM	0.379	< 0.85	Established
AWB–PDLS	0.125	< 0.85	Established
ELM–PDLS	0.258	< 0.85	Established

Author's own work.

As reported in Table 5, all four constructs satisfied the recommended criteria for internal consistency reliability and convergent validity. Cronbach's alpha values ranged from 0.887 to 0.906, while composite reliability values ranged from 0.917 to 0.930. All values exceeded the recommended threshold of 0.70 and remained below 0.95. The average variance extracted values ranged from 0.689 to 0.727, exceeding the recommended threshold of 0.50 for every construct. Full collinearity variance inflation factor values ranged from 1.064 to 1.353 and were substantially below the threshold of 3.3. Furthermore, all heterotrait-monotrait ratios were below the conservative threshold of 0.85, including the value of 0.184 between EI and PDLS. Therefore, the revised measurement model demonstrated satisfactory internal consistency reliability, convergent validity, discriminant validity, and acceptable collinearity. Table 6 shows the results of HTMT assessment of discriminant validity.

### 4.4 Hypothesis testing

The structural relationships were evaluated using the standardized path coefficients, standard errors, probability values, and effect sizes generated by WarpPLS 8.0. Hypothesis decisions were based on the direction and statistical significance of each estimated relationship. The analysis employed the Stable3 resampling procedure with 100 resamples. Consequently, the reported standard errors and probability values correspond to this procedure and should not be described as results obtained through conventional bootstrapping. Hypothesis testing results are displayed in Table 7 below. H1 proposed a positive association between EI and AWB. EI demonstrated a positive and statistically significant association with AWB (β = 0.354, *SE* = 0.046, *p* < 0.001, *f*^2^ = 0.125), supporting H1. This finding indicates that learners reporting stronger emotional competencies also tended to report higher levels of AWB. H2 proposed a positive association between EI and ELM. The association was positive and statistically significant (β = 0.331, *SE* = 0.046, *p* < 0.001, *f*^2^ = 0.134), supporting H2. This result indicates that greater EI was associated with stronger motivation to learn English. H3 proposed a positive association between AWB and ELM. AWB demonstrated a positive and statistically significant association with ELM (β = 0.229, *SE* = 0.047, *p* < 0.001, *f*^2^ = 0.080), supporting H3. Thus, learners experiencing more favorable academic functioning and well-being also tended to demonstrate stronger ELM. Because the research employed a cross-sectional design, these results represent statistical associations and do not establish causal or temporal relationships. H4 proposed that AWB provides an indirect pathway between EI and ELM. The specific indirect association was positive (β = 0.081, *SE* = 0.034, *p* = 0.008, *f*^2^ = 0.033), showing that H4 was provisionally supported. The magnitude of the indirect coefficient was consistent with the product of the coefficient linking EI with AWB (β = 0.354) and the coefficient linking AWB with ELM (β = 0.229).

**Table 7:** Hypothesis testing results.

Hypothesis	Structural relationship	β	Standard error	*p*-value	Effect size (*f*^2^)	Decision
H1	Association of EI with AWB	0.354	0.046	< 0.001	0.125	Supported
H2	Association of EI with ELM	0.331	0.046	< 0.001	0.134	Supported
H3	Association of AWB with ELM	0.229	0.047	< 0.001	0.080	Supported
H4	Indirect association of EI with ELM through AWB	0.081	0.034	0.008	0.033	Provisionally supported
H5	Moderating effect of PDLS on the association between AWB and ELM	0.180	0.047	< 0.001	0.029	Provisionally supported

Author's own work. β represents the standardized coefficient, and *f*^2^ represents the effect size. H1, H2, and H3 were supported by the direct association results. H4 represents the specific indirect association of EI with ELM through AWB. H5 represents the interaction involving AWB and PDLS in relation to ELM. H4 and H5 are provisionally supported because the analysis used the Stable3 procedure with 100 resamples and did not provide conventional bootstrapped confidence intervals. Accordingly, we interpret the simple slopes and conditional indirect-effect estimates descriptively.

Indirect association = 0.354 × 0.229 = 0.081.

The direct association between EI and ELM remained positive (β = 0.331, *p* < 0.001), while the total association was β = 0.412 (*p* < 0.001). This pattern is consistent with a complementary partial indirect pathway and provides provisional support for H4. However, because the analysis employed 100 Stable3 resamples and did not generate a conventional bootstrapped confidence interval, the result should not be regarded as inferential confirmation of mediation:


$$\begin{array}{c} f^{2}=\frac{\left(R^{2}\text{included }-\;R^{2}\text{excluded}\right)}{\left(1\;-\;R^{2}\text{included}\right)} \end{array}$$

H5 proposed that PDLS moderates the association between AWB and ELM. The interaction between AWB and PDLS was positive (β = 0.180, *SE* = 0.047, *p* < 0.001, *f*^2^ = 0.029), providing provisional support for H5. The positive coefficient indicates that the association between AWB and ELM became stronger as PDLS increased. The descriptive standardized simple slope was approximately 0.049 when PDLS was one standard deviation below its mean, 0.229 at its mean, and 0.409 when it was one standard deviation above its mean. These values describe an increasingly positive association across higher levels of PDLS. However, the simple slopes were derived algebraically from the standardized coefficients and were not accompanied by conventional bootstrapped confidence intervals. They are therefore interpreted descriptively and should not be regarded as inferential confirmation of moderation. The association between EI and ELM produced the largest direct-path effect size in the model (*f*^2^ = 0.134), followed by the association between EI and AWB (*f*^2^ = 0.125) and the association between AWB and ELM (*f*^2^ = 0.080). The indirect association through AWB had an effect size of 0.033, while the moderating effect of PDLS had an effect size of 0.029. The total association between EI and ELM was β = 0.412 (*p* < 0.001), with a total effect size of 0.167. The direct-effect results supported H1, H2, and H3. The coefficients and probability values generated through the Stable3 procedure provided provisional support for H4 and H5. However, the indirect and interaction findings require confirmation through conventional bootstrapping with more resamples and corresponding confidence intervals.

### 4.5 Mediation, moderation, and conditional indirect effects

The specific indirect association between EI and ELM through AWB was positive (β = 0.081, *SE* = 0.034, *p* = 0.008, *f*^2^ = 0.033). EI was positively associated with AWB (β = 0.354, *p* < 0.001), while AWB was positively associated with ELM (β = 0.229, *p* < 0.001). The direct association between EI and ELM remained positive (β = 0.331, *p* < 0.001). This pattern is consistent with a complementary partial indirect pathway and provides provisional support for H4 under the Stable3 procedure. The interaction between AWB and PDLS was also positive (β = 0.180, *SE* = 0.047, *p* < 0.001, *f*^2^ = 0.029), providing provisional support for H5. The descriptive standardized simple slope was approximately 0.049 when PDLS was one standard deviation below its mean, 0.229 at its mean, and 0.409 when it was one standard deviation above its mean. Based on the coefficient linking EI with AWB, the corresponding conditional indirect-effect point estimates were approximately 0.017 at low PDLS, 0.081 at its mean, and 0.145 at high PDLS. These results indicate that the positive association between AWB and ELM, as well as the corresponding indirect association involving EI, became stronger as PDLS increased. However, the simple slopes and conditional indirect-effect values were derived algebraically from the standardized coefficients and were not accompanied by conventional bootstrapped confidence intervals. They are therefore interpreted as descriptive estimates rather than inferentially confirmed conditional effects.

### 4.6 Consistency and reporting notes

All coefficients, probability values, effect sizes, and coefficients of determination reported in this section were checked against the revised WarpPLS output, Table 7, and Figure 2. EI was positively associated with AWB (β = 0.354, *p* < 0.001) and ELM (β = 0.331, *p* < 0.001), supporting H1 and H2. AWB was positively associated with ELM (β = 0.229, *p* < 0.001), supporting H3. The specific indirect association between EI and ELM through AWB was positive (β = 0.081, *p* = 0.008), providing provisional support for H4. The interaction between AWB and PDLS was also positive (β = 0.180, *p* < 0.001), providing provisional support for H5. The model explained 12.5% of the variance in AWB and 24.3% of the variance in ELM. All reported analyses used the same dataset of 435 usable responses and the same WarpPLS analytical procedure. Because H4 and H5 were assessed using 100 Stable3 resamples without conventional bootstrapped confidence intervals, their interpretation is restricted to provisional indirect and interaction associations.

## 5 Discussion

This study examined the relationships among EI, AWB, ELM, and PDLS in digitally supported English as a Foreign Language environments. The direct-effect results supported H1, H2, and H3. EI was positively associated with both AWB and ELM, while AWB was positively associated with ELM. The Stable3 results also indicated a positive indirect association between EI and ELM through AWB and a positive interaction between AWB and PDLS. These findings provided provisional support for H4 and H5. Collectively, the results indicate that learners' motivation to study English is associated with emotional competence, academic functioning, and perceived support within digital learning environments. Because the study employed a cross-sectional design, the findings represent statistical associations rather than evidence of causal or temporal relationships. In addition, the indirect and interaction findings require confirmation using conventional bootstrapping with a larger number of resamples and corresponding confidence intervals.

### 5.1 Emotional Intelligence (EI) and Academic Well-being (AWB) (H1)

EI was positively and significantly associated with AWB (β = 0.354, *p* < 0.001, *f*^2^ = 0.125), supporting H1. This result indicates that learners who reported stronger capacities to recognize, understand, and regulate their emotions also tended to report more favorable academic functioning and well-being. The finding is consistent with previous research linking emotional regulation to psychological functioning, academic adjustment, and resilience in educational settings (Huang et al., 2023; Mastrokoukou et al., 2024). In digitally supported English-learning environments, emotional competencies may help learners manage academic pressure, frustration, reduced face-to-face interaction, technological difficulties, and the greater autonomy required for online learning. EI may therefore function as an important personal psychological resource associated with learners' ability to respond constructively to academic demands. EI explained 12.5% of the variance in AWB, indicating meaningful but modest explanatory power. AWB is also likely to be associated with institutional support, peer relationships, academic workload, instructional practices, family conditions, and broader educational experiences. Because the research design was cross-sectional, the positive coefficient should be interpreted as an association and not as evidence that EI causally improves AWB.

### 5.2 Emotional Intelligence (EI) and English Learning Motivation (ELM) (H2)

EI demonstrated a positive and statistically significant association with ELM (β = 0.331, *p* < 0.001, *f*^2^ = 0.134), supporting H2. This relationship produced the largest direct-path effect size in the structural model. Learners reporting stronger EI also tended to demonstrate greater willingness to invest effort, persist when learning became difficult, and remain committed to improving their English proficiency. This finding is consistent with research emphasizing the relevance of emotional regulation to learner engagement, persistence, and motivation (Macklem, 2015; Xu et al., 2026). The relationship may be particularly important in digitally supported English-learning environments, where learners often assume greater responsibility for organizing their learning and may encounter frustration, anxiety, technological difficulties, or limited direct interaction with instructors. From the perspective of Self-Determination Theory, EI may represent a psychological resource associated with learners' ability to regulate negative emotions and maintain goal-directed learning behavior under conditions requiring greater autonomy and self-regulation. Nevertheless, ELM is also associated with instructional quality, teacher and peer support, technological accessibility, learner confidence, and the perceived value of English proficiency. Because the data were cross-sectional, the result indicates an association and does not establish that EI causes stronger ELM.

### 5.3 Academic Well-being (AWB) and English learning motivation (H3)

AWB was positively and significantly associated with ELM (β = 0.229, *p* < 0.001, *f*^2^ = 0.080), supporting H3. Learners reporting greater satisfaction, emotional balance, academic engagement, and constructive responses to academic demands also tended to demonstrate stronger motivation to learn English. This result is consistent with previous research associating favorable academic experiences with engagement and sustained participation in educational activities (Sá, 2023). In digitally supported English-learning environments, positive academic functioning may help learners sustain effort when they encounter complex learning activities, technological difficulties, or reduced direct interaction with instructors and classmates. Nevertheless, the relatively modest effect size indicates that AWB represents only one of several factors associated with ELM. Emotional competencies, instructional practices, teacher and peer support, technological accessibility, learner confidence, and the perceived value of English proficiency may also explain motivational differences. Therefore, the result supports the proposed positive association while recognizing the multidimensional nature of ELM. Because the research employed a cross-sectional design, the finding should not be interpreted as evidence that AWB causally increases ELM.

### 5.4 Mediating role of Academic Well-being (AWB) (H4)

The specific indirect association between EI and ELM through AWB was positive (β = 0.081, *SE* = 0.034, *p* = 0.008, *f*^2^ = 0.033). EI was positively associated with AWB, and AWB was positively associated with ELM. The direct association between EI and ELM also remained positive after AWB was included in the model. This pattern is consistent with a complementary partial indirect pathway and provides provisional support for H4 under the Stable3 procedure. The result suggests that EI may be associated with ELM partly through its positive association with learners' academic functioning and well-being. Learners who are better able to recognize, understand, and regulate their emotions may experience more favorable academic conditions, which may subsequently be associated with greater persistence and commitment to English learning. AWB may therefore represent a relevant psychological pathway connecting emotional competence with ELM. This interpretation remains provisional because the analysis employed 100 Stable3 resamples and did not generate a conventional bootstrapped confidence interval for the indirect association. Accordingly, the finding should not be presented as inferential confirmation of mediation. A future analysis using conventional bootstrapping with a substantially larger number of resamples and a corresponding confidence interval is required to establish the precision and robustness of the indirect association.

### 5.5 Moderating role of Perceived Digital Learning Support (PDLS) (H5)

The interaction between AWB and PDLS was positive (β = 0.180, *SE* = 0.047, *p* < 0.001, *f*^2^ = 0.029), providing provisional support for H5 under the Stable3 procedure. The positive interaction coefficient indicates that the association between AWB and ELM became stronger as PDLS increased. The descriptive standardized simple slope was approximately 0.049 when PDLS was one standard deviation below its mean, 0.229 at its mean, and 0.409 when it was one standard deviation above its mean. This pattern suggests that AWB had only a weak positive association with ELM at lower levels of PDLS, whereas the association became progressively stronger at average and higher levels of support. The finding is consistent with Abubakar et al. (2026), who emphasized the importance of supportive educational conditions for learner engagement. Access to appropriate digital resources, useful feedback, clear guidance, and timely technological assistance may create conditions in which favorable academic experiences are more strongly associated with sustained motivation. PDLS may therefore provide a contextual condition that strengthens the motivational relevance of AWB. The heterotrait-monotrait ratio between EI and PDLS was 0.184, supporting the empirical distinction between the two constructs. Nevertheless, the simple-slope values were derived descriptively from the standardized coefficients and were not accompanied by conventional bootstrapped confidence intervals. Therefore, the interaction pattern should be interpreted as provisional rather than as inferential confirmation of moderation.

### 5.6 Integrated interpretation and theoretical contribution

The revised findings indicate that ELM in digitally supported English as a Foreign Language environments is associated with emotional, psychological, and digital-contextual conditions. EI was positively associated with AWB and ELM, while AWB was positively associated with ELM. These direct-effect results supported H1, H2, and H3. The Stable3 results also indicated a positive indirect association between EI and ELM through AWB. In addition, the positive interaction between AWB and PDLS indicated that the association between AWB and ELM became stronger as perceived support increased. These results provided provisional support for H4 and H5. However, the absence of conventional bootstrapped confidence intervals prevents the indirect and interaction findings from being treated as inferentially confirmed mediation and moderation effects. The study contributes to the literature by integrating EI, AWB, ELM, and PDLS within one structural framework. This integration demonstrates that learner motivation is associated with both internal psychological resources and external digital-learning conditions. The indirect pattern suggests that AWB may represent a psychological pathway connecting EI with ELM. The interaction pattern further suggests that PDLS may strengthen the motivational relevance of favorable academic functioning. The model explained 12.5% of the variance in AWB and 24.3% of the variance in ELM. The corresponding Stone-Geisser predictive-relevance coefficients were 0.126 and 0.244. These values indicate acceptable predictive relevance, although substantial proportions of variance remained unexplained. The model should therefore be understood as a focused explanation of selected emotional, psychological, and contextual associations rather than a complete account of AWB or ELM. The theoretical contribution is strongest for the three direct associations, which were supported by the revised structural results. The indirect and interaction patterns extend the proposed framework but remain provisional until they are confirmed through conventional bootstrapping with a larger number of resamples and corresponding confidence intervals.

## 6 Conclusion

This study examined the relationships among EI, AWB, ELM, and PDLS in digitally supported English as a Foreign Language environments. Data from 435 respondents were analyzed using partial least squares structural equation modeling through WarpPLS 8.0. The direct effect results supported H1, H2, and H3. EI was positively associated with AWB (β = 0.354, *p* < 0.001) and ELM (β = 0.331, *p* < 0.001). AWB was also positively associated with ELM (β = 0.229, *p* < 0.001). The Stable3 output indicated a positive specific indirect association between EI and ELM through AWB (β = 0.081, *p* = 0.008). It also indicated a positive interaction between AWB and PDLS in relation to ELM (β = 0.180, *p* < 0.001). These results provided provisional support for H4 and H5. However, because the analysis employed 100 Stable3 resamples and did not generate conventional bootstrapped confidence intervals, the results should not be regarded as inferential confirmation of mediation or moderation. The measurement model demonstrated satisfactory quality. Indicator loadings ranged from 0.778 to 0.880, Cronbach's alpha values ranged from 0.887 to 0.906, composite reliability values ranged from 0.917 to 0.930, and average variance extracted values ranged from 0.689 to 0.727. All heterotrait-monotrait ratios were below 0.85, supporting discriminant validity. Full collinearity variance inflation factor values were also below 3.3. The model explained 12.5% of the variance in AWB and 24.3% of the variance in ELM. These findings indicate that emotional competence, academic functioning, and perceived digital support provide complementary explanations of learners' motivation. Nevertheless, the cross-sectional design does not establish temporal ordering or causality. The direct coefficients represent statistical associations, while the indirect and interaction patterns require confirmation using conventional bootstrapping with a larger number of resamples and corresponding confidence intervals.

### 6.1 Practical mechanisms and illustrative scenarios

The findings suggest that ELM may be strengthened by coordinated attention to EFL learners' emotional competencies, academic experiences, and digital learning conditions. Educational institutions could introduce activities that develop emotional awareness, emotional regulation, resilience, and constructive responses to academic pressure. Relevant practices may include reflective learning exercises, structured peer support, academic counseling, and guidance on managing frustration and anxiety during EFL learning. The positive association between EI and AWB indicates that emotional development may correspond with more favorable academic functioning. Instructors could support this relationship by establishing attainable learning objectives, providing constructive feedback, maintaining manageable workloads, encouraging meaningful interaction, and recognizing learner progress. These practices may help learners sustain effort and persistence during demanding English learning activities. The provisional indirect pattern suggests that AWB may represent a psychological pathway connecting EI with ELM. Accordingly, emotional development initiatives may be more useful when accompanied by efforts to improve learners' academic experiences. This implication remains tentative because the indirect association was not evaluated using a conventional bootstrapped confidence interval. The provisional interaction pattern further suggests that digital support may strengthen the motivational relevance of AWB. Institutions should provide accessible learning materials, reliable platforms, clear technical guidance, timely instructor feedback, and responsive assistance when learners experience technological difficulties. For example, learners with favorable AWB may demonstrate stronger motivation when digital resources are accessible and instructional feedback is timely. Conversely, unreliable platforms or inadequate support may restrict learners' ability to translate favorable academic experiences into sustained motivation. These recommendations should be implemented as complementary practices rather than isolated interventions. Emotional development programmes may provide limited benefits when learners encounter poorly designed digital environments, while technological investments may provide limited motivational value when learners experience emotional strain or unfavorable academic conditions. Because the indirect and interaction results remain provisional, these recommendations should be evaluated through carefully designed institutional interventions rather than treated as confirmed causal mechanisms.

### 6.2 Theoretical, practical, and societal implications

From a theoretical perspective, the study provides reliable evidence for the direct associations among EI, AWB, and ELM. EI was positively associated with both AWB and ELM, while AWB was positively associated with ELM. These findings support the integration of emotional competence and academic psychological functioning within explanations of motivation in digitally supported English learning. The Stable3 results additionally suggest that AWB may provide an indirect pathway connecting EI with ELM. The interaction result also suggests that the strength of the association between AWB and ELM may vary according to learners' PDLS. These theoretical extensions remain provisional because conventional bootstrapped confidence intervals were unavailable. From a practical perspective, educators should not treat learners' emotional development, academic functioning, and digital learning conditions as unrelated concerns. Institutions may support motivation by combining emotional regulation activities with accessible digital resources, clear instructional guidance, interactive learning content, and timely feedback. Improvements in digital infrastructure should be accompanied by instructional and psychological support so that technological accessibility contributes to meaningful learning experiences. From a societal perspective, accessible and supportive digital learning environments may contribute to more equitable opportunities for language learning. Learners who lack reliable technological assistance, appropriate resources, or clear digital guidance may be less able to benefit from favorable academic experiences. Educational policies should therefore consider technological access, instructional support, emotional competence, and psychological well-being jointly when developing digitally supported language learning initiatives.

### 6.3 Originality and contribution

This study contributes to research on digital English as a Foreign Language learning and educational psychology in four respects. First, it integrates EI, AWB, ELM, and PDLS within a single structural framework. This approach provides a more comprehensive explanation than examining each construct as an isolated predictor. Second, the study provides reliable evidence that EI is positively associated with AWB and ELM. It also demonstrates a positive association between AWB and ELM. These findings clarify the interconnected roles of emotional competence and academic psychological functioning in digitally supported English learning. Third, the Stable3 results identify a positive indirect pattern involving EI, AWB, and ELM. EI remained positively associated with ELM after AWB was included in the model, while the direct and indirect coefficients operated in the same direction. This pattern is consistent with a complementary partial indirect pathway, although confirmation through conventional bootstrapping remains necessary. Fourth, the positive interaction involving PDLS suggests that the association between AWB and ELM becomes stronger as perceived digital support increases. This finding highlights the possible contextual importance of accessible resources, feedback, guidance, and technological assistance. However, the interaction should be regarded as provisional until its confidence interval and simple slopes are evaluated through conventional bootstrapping. The revised measurement analysis also established satisfactory indicator reliability, internal consistency reliability, convergent validity, and discriminant validity. In particular, the heterotrait-monotrait ratio of 0.184 between EI and PDLS confirms that the two constructs were empirically distinguishable. The study therefore contributes an integrated measurement and structural framework for examining how emotional competence, academic functioning, and digital support are associated with ELM.

### 6.4 Limitations and future research

Several limitations should be considered when interpreting the findings. First, the study employed a cross-sectional design in which all variables were measured at a single point in time. Temporal ordering and causal relationships cannot therefore be established. Although the structural model specifies theoretically directed relationships, the estimated coefficients represent associations consistent with the proposed framework. Longitudinal, experimental, and repeated measures research would provide stronger evidence concerning the direction and stability of these relationships. Second, the data were obtained through a single self-report questionnaire completed by the same respondents. The findings may therefore be affected by common method variance, social desirability, individual response tendencies, or differences in participants' interpretations of the items. Although all full collinearity variance inflation factor values were below 3.3, future studies could combine self-reported measures with teacher evaluations, behavioral indicators, learning platform records, performance measures, or longitudinal observations. Third, the WarpPLS analysis employed the Stable3 procedure with 100 resamples. The output provided standard errors and probability values but did not provide conventional bootstrapped confidence intervals for the indirect association, interaction association, simple slopes, or conditional indirect effects. The algebraically derived simple slopes and conditional indirect-effect values were therefore presented only as descriptive estimates. Future research should use conventional bootstrapping with a substantially larger number of resamples and report confidence intervals for all direct, indirect, interaction, and conditional indirect associations. Fourth, the model explained 12.5% of the variance in AWB and 24.3% of the variance in ELM. Although the corresponding predictive relevance coefficients exceeded zero, substantial proportions of variance remained unexplained. Future research could examine additional learner, instructional, social, and institutional factors, including self-efficacy, language learning anxiety, teacher support, peer interaction, academic pressure, instructional quality, and technological accessibility. Fifth, college and university learners were combined in the pooled sample. This approach increased sample diversity but did not establish that the measurement properties or structural relationships were equivalent across educational levels. The study did not conduct a formal Measurement Invariance of Composite Models assessment or multigroup analysis. Future research should establish measurement invariance before comparing structural associations across college and university learners. Finally, the sample was obtained through convenience sampling from participating educational institutions in Henan Province. Consequently, the findings should not be generalized automatically to learners in other regions, institutional settings, age groups, or cultural contexts. Replication using probability-based sampling, broader geographical coverage, independent samples, and different educational contexts would strengthen the external validity of the model.

## Data Availability

The original contributions presented in the study are included in the article/supplementary material, further inquiries can be directed to the corresponding authors.
